# Characterizing Trends in the Use of Food Donations and Other Food-Related Community-Based Social Assistance Programs in a Cohort of New Food Bank Users in Quebec, Canada

**DOI:** 10.3389/ijph.2024.1605833

**Published:** 2024-02-09

**Authors:** Elsury Johanna Pérez, Mabel Carabali, Geneviève Mercille, Marie-Pierre Sylvestre, Federico Roncarolo, Louise Potvin

**Affiliations:** ^1^ School of Public Health, Department of Social and Preventive Medicine, Université de Montréal, Montréal, QC, Canada; ^2^ Centre de Recherche en Santé Publique (CReSP), Université de Montréal & CIUSSS du Centre-Sud-de-l’île-de-Montréal, Montréal, QC, Canada; ^3^ Chaire de Recherche du Canada sur les Approches Communautaires et Inégalités de Santé (CACIS), Université de Montréal, Montréal, QC, Canada; ^4^ Department of Epidemiology, Biostatistics and Occupational Health, School of Population and Global Health, McGill University, Montréal, QC, Canada; ^5^ Department of Nutrition, Faculty of Medicine, Université de Montréal, Montréal, QC, Canada; ^6^ Centre de Recherche du Centre Hospitalier de l’Université de Montréal (CRCHUM), Montréal, QC, Canada

**Keywords:** alternative food sources, food banks, food assistance, community-based food programs, Canada, food access, Quebec

## Abstract

**Objective:** To characterize 12-month trends in the use of food donations and other food-related community-based social assistance programs (CB-SAPs) during the first year following the enrollment of new food bank (FB) users in Quebec, Canada.

**Methods:** A cohort of 1,001 newly registered FB-users in Quebec from the Pathways Study were followed-up during 12-month following baseline assessment. Outcomes were monthly use of food donations and other food-related CB-SAPs. Main predictors were alternative food source utilization (AFSU) profiles: 1) exclusive-FB-users; 2) FB+fruit/vegetable-market-users; and 3) Multiple/diverse-AFS-users. Covariates included sociodemographic characteristics, health status, and major life events. We fit Bayesian hierarchical mixed-effect models, accounting for spatial clustering, temporal correlation, and censoring.

**Results:** We observed an overall downward trend of food donation use among study completers (*n* = 745). Each AFSU profile had a distinctive monthly trend of food donation use, but probabilities of use across the three profiles overlapped, between 44% and 55%. The use of other food-related CB-SAPs was low and not correlated with AFSU profiles.

**Conclusion:** *De novo* FB-users use food donations in different ways over time according to specific contextual AFSU profiles.

## Introduction

The use of food banks (FBs) has been increasing in Canada since their conception in the 1980s [1], illuminating the pressing problem of hunger even among the richest countries [2]. In 2022, in ten Canadian provinces, 4.2% of households (i.e., 1.5 million individuals) experienced severe food insecurity, defined as the reduction in the amount of food consumed due to lack of financial resources [3]. FBs are organizations which collect surplus, wasted, or donated food and distribute it in the form of food baskets. FBs have become an important alternative food source -AFS- (i.e., ways through which people can procure food outside the regular food system) for people who are experiencing or are at risk of experiencing severe food insecurity [4–9]. In Québec, most FBs are integrated in community organizations also offering other food-related community-based social assistance programs (CB-SAPs) (i.e., collective kitchens, collective gardens, collective food-buying groups, food sales service, and community meals) for people in need [10]. Little is known about how FB-users use food donations and other food-related CB-SAPs over time.

FB-users are a heterogenous population with diverse socioeconomic backgrounds and needs [11, 12]. To feed themselves and their household, FB-users take advantage of diverse strategies to exploit their limited resources and overcome structural barriers such as transport limitations, high food prices, and limited food access [9, 11, 13, 14]. These strategies classified into coping and adapting strategies are implemented by people with different capacities and assets in specific contexts [15]. Coping strategies offset shocks that jeopardize food availability [15]. One important coping strategy used by FB-users is the utilization of multiple AFSs (including diverse types of FBs, fruit and vegetable (F&V) markets, domestic food production, etc.) with different frequencies and travel times [16]. Our research group previously identified three profiles of alternative food source utilization (AFSU) among new FB-users that vary across urban, suburban, and rural settings: FB-exclusive-users, FB+F&V-market-users, and multiple/diverse-AFS-users. The study showed that in rural settings, couples with or without children are more likely to be multiple/diverse-AFS-users, whereas in urban settings, more educated households are more likely to belong in this profile [17]. In the long-term, FB-users employ adapting strategies which are coping strategies that become integrated into the normal cycle of households activities [15].

Studies have indicated that some FB-users may use FBs sporadically due to unexpected events or emergencies and others may become long term users [18–23]. Using data from the Greater Vancouver Food Bank from 1992 through 2017, Black et al. (2020) identified three patterns of FB-users that were correlated with sociodemographic and health-related characteristics: transitional-users (91%) who visited FBs on average seven times over 2 months and then stopped; episodic-users (7%) who used FB for an average of 8.6 years with a mean number of visits of 100 over the period; and chronic-users (1.5%) who visited FB at least 200 hundred times over several years (mean > 13 years) [24]. Understanding the link between short- and long-term strategies to cope with food insecurity among FB-users could help to better understand why it is that some food insecure households become food secure, whilst others do not, but it has not yet been documented in quantitative studies [25, 26]. This study aims to characterize 12-month trends in the use of food donations and other food-related CB-SAPs during the first year following enrollment of new FB-users in Quebec, Canada.

## Methods

### Design, Sampling, and Data Source

This longitudinal prospective study uses data from the baseline (t_0_) and 1-year follow up (t_1_) from the *Pathways Study*. Our population consists of newly enrolled FB-users in urban, semiurban, and rural settings of Quebec, Canada. Baseline data were collected from September 2018 to January 2020, from 1,001 newly registered FB-users in 106 community organizations offering food donations in four regions of Quebec (Montréal, Lanaudière, Mauricie-Centre-du-Québec, Estrie). These organizations regularly distribute food baskets to people in need, but they vary in their services (e.g., size and quality of food baskets), structure, and operations depending on their resources and specific context. Some of them also provide other food-related CB-SAPs (e.g., collective kitchens, food-buying groups, food sales service). Follow-up data were collected in a window of 11–13 months after the baseline interview [27]. Inclusion criteria were: 1) FB-users who used food donations for the first time within a maximum of 6 months prior to recruitment, 2) between 18 and 63 years of age, and 3) being able to communicate in French or English. People living with a person who was already enrolled in the study and persons experiencing homelessness were excluded. Details of the Pathways Study are presented elsewhere [27].

Baseline data were collected by trained interviewers via face-to-face interviews conducted at the community organizations where the food donation occurs. COVID-19 related lockdowns were in place in the middle of the second wave of interviews, forcing the second half of follow-up interviews to be conducted over the phone or online.

### Variables

#### Outcome

*Trends in the use of food donations and other food-related CB-SAPs during the 12* *months following FB enrollment.* The use of food donations and other food-related CB-SAPs over 12-month period was ascertained by asking participants whether they used (yes/no) these programs in each of the 12 months prior to the follow-up interview. The use of other food-related CB-SAPs refers to the use of at least one of the following CB-SAPs: including collective kitchens, collective gardens, food buying groups, food sales service, and community meals. We grouped these programs due to the limited usage observed among participants (between 3.4% for community garden and 10% for community meals).

#### Main Independent Variable

**FIGURE 1:**
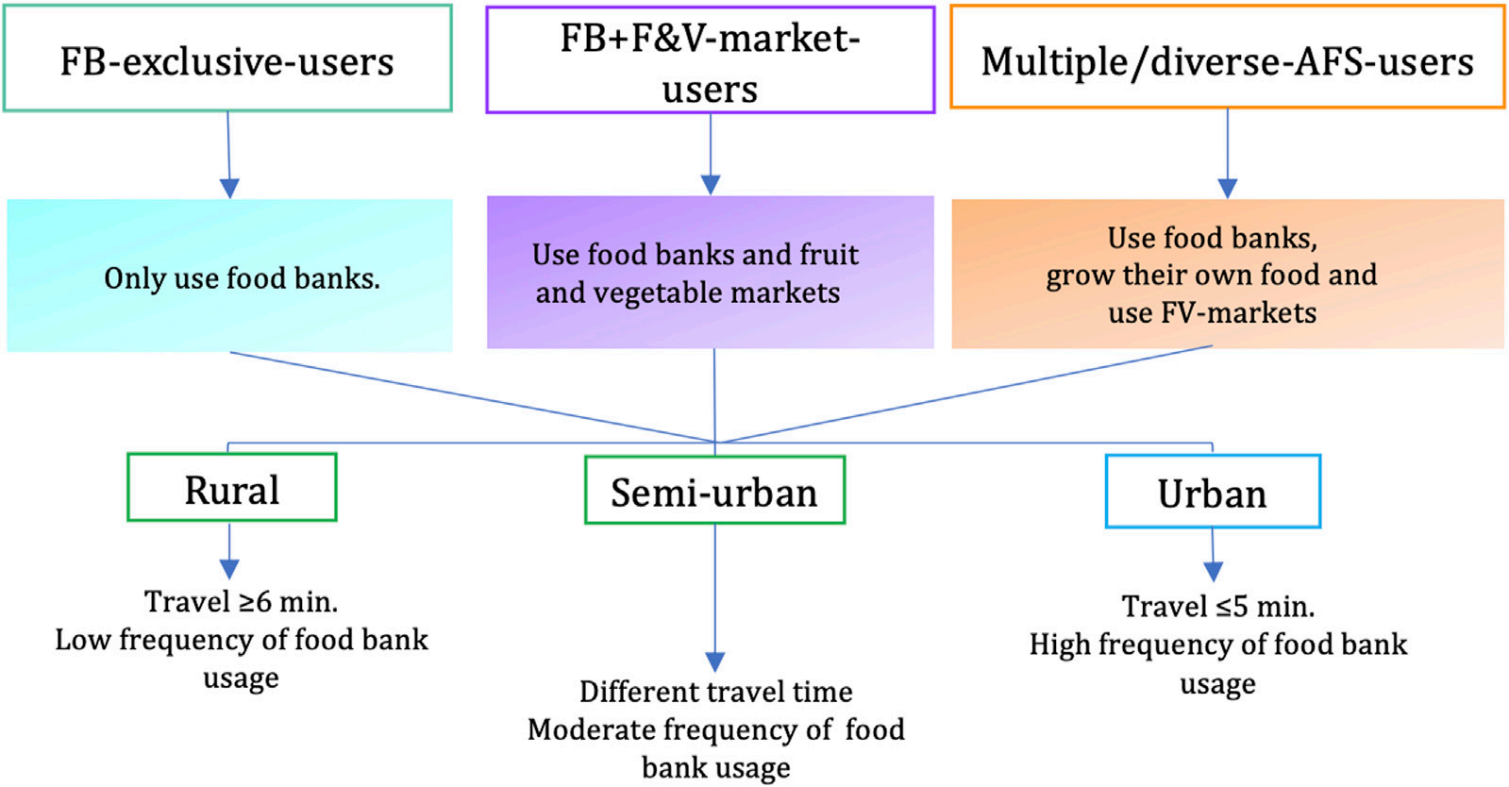
Alternative food source utilization profiles of newly enrolled food bank users in Quebec, Canada (2018–2021).

*Alternative food source utilization profiles.* At baseline, participants were classified into three distinctive profiles: FB-exclusive-users, FB+F&V-market-users, and multiple/diverse-AFS-users. These profiles were created to reflect on the differential characteristics and food-acquisition practices of participants study, as well as their differences across settings (e.g., urban, semiurban, and rural). AFSU profiles were created using five items related to AFS utilization: 1) Type of food bank used (i.e., Capacity-Building Programs [CBP-FBs] were those community organizations offering food donations and other CB-SAPs, and Food Donations [FD-FBs] were community organizations providing only food) [12]; 2) Use of F&V markets during the summer; 3) Growing one’s own food during the summer; 4) Frequency of food donations use; and 5) Travel time to the grocery store. An overview of these profiles is presented in Figure 1. Further details about item assessment and profiles computation were summarized in a prior paper [17].

#### Covariates

To account for the nested and longitudinal structure of the data, as well as to adjust the variance, we included variables related to the setting, size of community organization from which participants were recruited, *COVID-19* measures, and time with the following parameterization: *Setting* indicates the location of the community organisation where participants were recruited. It was defined as urban, semi-urban, and rural using Statistics Canada’s Census Metropolitan Areas (CMAs) and Census Agglomerations (CAs) categorization [28], in conjunction with Regional County Municipalities’ development plans, used by administrative regions in Quebec. Urban settings are characterized by a large and diverse population, building density and mixed land uses. Semi-urban settings are characterized by a less diverse population and are closer to rural settings than to the urban centres. Rural settings are characterized by low population density and a lack of diversity in land use. Details about the classification of this variable are presented elsewhere [27]. *Size of* c*ommunity organization* is a variable created based on the number of participants recruited from each community organization to account for any deviation from the proportionality of the sampling. *COVID-19 measures* indicates whether the participant completed the follow-up interview before or after COVID-19 lockdown measures in Quebec were in place, because the COVID-19 pandemic could have an impact on follow up of participants, covariates, and the use of CB-SAPs.

The selection of the other covariates was based on prior theoretical and empirical research about AFSU profiles and trends in food donation use [24, 29–32]. Baseline covariates included gender (male, female, or other), age in years (continuous), race/ethnicity, country of birth, household composition, household educational level, and length of FB use time prior to the study. Validated questions from the Canadian Community Health Survey and Canada Census were used to assesses all sociodemographic variables. *Race/ethnicity* was assessed by asking participants to indicate to which racial or cultural group they belong (White, South Asian, Chinese, Black, Filipino, Latin American, Arab, Southeast Asian, Korean, Japanese, or other). This variable was dichotomized into Whites and other due to data sparsity for non-White groups. *Country of birth* as self-reported by the participants, was dichotomized into Canada and other. *Household composition* was determined by asking participants to indicate which of type of household best described the one they lived in. This variable was grouped into three categories: single-parent household, couple with/without children, and single (i.e., individuals living alone or with others but without a child under their care or a marital or common-law relationship). *Household educational level* was determined by the highest educational level achieved by the respondents or their partner. This variable was dichotomized: secondary studies (11th grade) or less, and post-secondary studies. *Length of FB use time before the study* represents the time in months which had elapsed between the first time the participant visited the community organization offering the FB service and their study entry, determined by asking: “*How long you had been visiting this organization?*”. Further, we dichotomized the answers into ≤1 and 2–6 months.

Annual household income, presence of major life events, and physical and mental health were assessed at the baseline (t_0_) and at follow-up (t_1_). Participants were asked to indicate their *total annual household income from all sources before taxes* in the previous year. It was collected as a categorical variable with twelve levels in Canadian Dollars (CAN$), going from no income to CAN$≥100,000. Given that more than half of the participants reported total annual household income lower than CAN$14,999, this variable was dichotomized into CAN$≤14,999 and CAN$≥15,000.

*Major life events during the past 12* *months* were measured using Brugha and Cragg’s list of threatening events LTE-Q [33], which has been used in epidemiological studies and validated among divers populations [34, 35]. This scale includes 12 questions about major negative events such as serious illness, death of a loved one, separation, or loss of a job, that may influence mental health. It varies from 0 to 12. This variable was classified into two-or-less major life events and ≥3 major life events, given that the COVID-19 pandemic can be considered as a major event and increase the probability of experiencing more than one major life event during the study period.

*Physical and mental health* was measured using the 12-item Short Form Health Survey (SF-12), a valid and reliable tool to assess an individual’s perception about their own physical and mental health [36, 37]. The tool’s eight domain scores (physical functioning, role physical, role emotional, body pain, general health perceptions, vitality, social functioning, and mental health) were summarized into two components: Physical Component Summary (PCS) and Mental Component Summary (MCS). Both were standardized according to Ware et al. 2002, with scores > 50 indicating better than sample average health [38].

We included an indicator variable, *time,* indicating the month (from 1 to 12) after enrollment in the study for which the participant provided the information.

### Statistical Analysis

Descriptive statistics, including median and interquartile ranges (IQRs) for continuous variables and percentage for categorical variables, were calculated for all observations.

We used a Bayesian hierarchical mixed model logistic regression to assess the overall use of food donations and other food-related CB-SAPs trends associated to AFSU profiles over time. AFSU profile, sociodemographic covariates, mental health (centered at the mean), physical health and size of the community organization (as log-transformed variables) were modelled as fixed effects.

To identify the trends of use by the AFSU profiles we included a random effect interaction term of AFSU profiles and time, where time was parameterized as continuous with an autoregressive correlation of a first-order (AR1) structure to account for the repeated measures and autocorrelation within the same profile over time. To account for the nested structure (i.e., each participant had twelve observations, one for each month, T_1_ to T_12_) and any residual autocorrelation between measures over time, we included random effects variables for the *User* (i.e., the ID variable for each participant) and the time variable, both parameterized as independent and identically distributed (i.i.d) random effects. A version of the final model is presented in Eq. 1 and details of explored models are presented in Supplementary Tables S1, S2.

$$Y=logit\left(p_{it}\right)=\log\left(\frac{p_{it}}{1-p_{it}}\right)=\beta_{0}+X_{i}^{\prime }\;\beta_{x1}{+Z}_{it}^{\prime }\;\beta_{x}+u_{i}+w_{t}+z_{i}^{X*t},$$ (1)

Where $p_{it}$ is the probability of use of food donations or other food-related CB-SAP for an individual i (*i*_*1,*_ *i*_*n*_) at time t (*t*_*1, …*_ *t*_*12*_) and the exponentiated beta coefficient is the odds ratio (OR) for the use of food donations or other food-related CB-SAP; $\beta_{0}$ is the model’s intercept and $X_{i}^{\prime }\;\beta_{1}$ is the individual fixed effect for the AFSU profile; $Z_{it}^{\prime }\;\beta_{x}$ is a vector of the individual or setting fixed effects covariates described above; $u_{i}$ and $w_{t}$ are the i.i.d random effects for the user (to account for the correlated data between observation of the same individual) and time (to capture any residual temporal effect) respectively; and $z_{i}^{X^{\prime }*t}$ is the interaction term between the AFSU profile and time as AR1 to determine the trend of food donations or other food-related CB-SAP use over the study period. Results are transformed using the inverse logit function $\left(\frac{1}{1+\exp\left(-p_{it}\right)}\right)$ to obtain and present probabilities of food-related CB-SAP use by AFSU profiles over time.

### Missing Data

The distribution of missing data is presented in Supplementary Table S3. Although the maximum percentage of missing values of any covariate at baseline was low (<3.2%), we imputed missing data on covariables (e.g., annual household income, age, major life events, health status, race, and country of birth) before performing Bayesian hierarchical mixed models, using multiple imputation by chained equations (MICE) (100 sets).

Given that the losses to follow-up in this study may be influenced by participants’ sociodemographic characteristics, AFSU profiles, and use of CB-SAPs, we used inverse probability of censoring weighting (IPCW) to account for the potential differential attrition. With the IPCW we upweighted the observations from participants who remained in the study to account for those who were lost to follow-up (i.e., missing at 1 year follow-up), reconstructing the study population that we would have observed without attrition [39]. Thus, IPCW represents the inverse probability of remaining in the study at t_1_, which means 12 observations of CB-SAP use before the follow-up. Weights were estimated using logistic regression including all covariates before to be included in the final model. We used stabilized weights and compare the results from the IPCW model with results from the unweighted model.

### Model Selection

To assess model fit we specified different model regressions using different setting parameters, including them as random effects and different autoregressive formulations for the time as the residual and the interaction term. Model selection was informed by Deviance Information Criterion (DIC), the Watanabe-Akaike information criterion (WAIC) and the precision of the hyperparameters for the random effects. To obtain the posterior distribution of the estimates and the respective 95% Credible Intervals (95% CrI), all analyses were performed using the Integrated Nested Laplace Approximation (INLA) package with non-informative priors [40] in R-Studio version 4.2.1 (R Core Team. R, 2019).

## Results

Among the 1,001 participants enrolled with baseline data, 745 (74.4%) provided data at *t*_*1*_. Compared to participants with data in the two waves, a higher proportion of those lost-to-follow-up were white Canadian single males, who lived in urban settings and reported lower household education level and income. A complete description of the Pathways Study sample and sociodemographic differences among participants across settings have been previously outlined [41]. FB-exclusive-users presented the highest proportion of lost-to-follow-up going from 35.5% to 29.1%, while the proportion of the other profiles increased (5.2% and 1.2%) at *t*_*1*_ (Supplementary Table S4).

**FIGURE 2:**
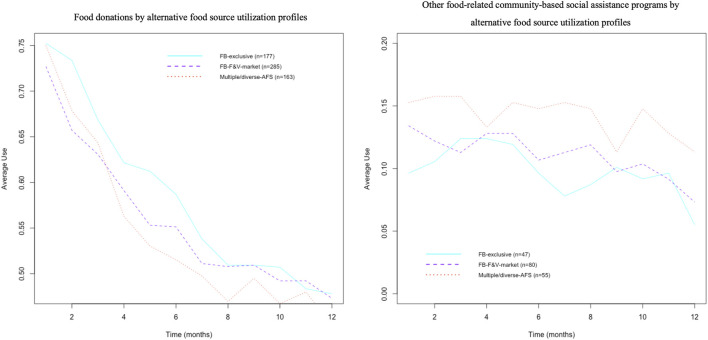
Unadjusted trends (proportion) in the use of food donations and other food-related community-based social assistance programs among newly enrolled food bank users in Quebec, Canada (2018–2021).

The distribution of AFSU profiles by sociodemographic characteristics is presented in Table 1. At *t*_*1*_, 29.1% of participants were classified as FB-excusive-users (*n* = 217), 43.9% were FB+F&V-market-users (*n* = 327), and 27% were multiple/diverse-AFS-users (*n* = 201). The full description of AFSU profiles and their socioeconomic differences in urban, semi-urban, and rural settings have been presented in a prior paper [17]. In both waves, most of the participants in all AFSU profiles were white Canadian single women, reported an annual household income CAN$≤14,999, and did not have post-secondary education among all profiles. However, the proportion of participants that reported having an annual household income CAN$ ≥15,000 increased in all groups at *t*_*1*_. More than 81% of participants reported having used food donations at least once after the baseline and the mean use of this program in all AFSU profiles presented a downward trend going from 75% to 50%. In contrast, less than 20% of participants used other food-related CB-SAPs and the mean use of these programs in all AFSU profiles did not change over time (Figure 2).

**TABLE 1:** Characteristics of participants at baseline and follow-up by alternative food source utilization profiles on monthly use of food donations among newly enrolled food bank users in Quebec, Canada (2018–2021).

Characteristic	Baseline			Follow-up		
	Alternative food source utilization profiles					
	FB-exclusive- users (*n* = 308)	FB = F&V- market-users (*n* = 426)	Multiple/diverse-AFS-users (*n* = 267)	FB-exclusive- users (*n* = 217)	FB = F&V-market-users (*n* = 327)	Multiple/diverse-AFS-users (*n* = 201)
Age, y, median (IQR)	41.0 (31.0, 53.2)	41.0 (32.0, 51.0)	40.0 (32.0, 49.5)	42.0 (31.5, 54.0)	40.0 (32.5, 51.0)	40.0 (32.5, 51.0)
Gender (%)						
Female	172 (55.8)	236 (55.4)	204 (76.4)	132 (60.8)	192 (58.7)	161 (80.1)
Male	136 (44.2)	190 (44.6)	63 (23.6)	85 (39.2)	135 (41.3)	40 (19.9)
Country of birth (%)						
Canada	256 (83.1)	286 (67.1)	233 (87.3)	170 (78.3)	214 (65.4)	175 (87.1)
Other	52 (16.9)	140 (32.9)	34 (12.7)	47 (21.7)	113 (34.6)	26 (12.9)
Race (%)						
White	247 (80.32)	276 (64.8)	227 (85.0)	167 (77.0)	206 (63.0)	172 (86.0)
Other	61 (19.8)	150 (35.2)	40 (15.0)	50 (23.0)	121 (37.0)	28 (14.0)
Mental health, median (IQR)	40.7 (30.5, 48.3)	40.7 (32.6, 50.5)	41.9 (32.6, 49.9)	42.9 (34.4, 51.6)	43.9 (35.1, 52.6)	43.3 (35.8, 52.7)
Physical health, median (IQR)	47.4 (36.1, 55.1)	48.6 (35.4, 56.6)	49.8 (35.6, 57.5)	47.6 (37.3, 54.8)	49.7 (36.1, 56.9)	50.8 (34.4, 57.2)
Major life events, median (IQR)	3.0 (2.0, 5.0)	3.0 (1.0, 4.0)	4.0 (2.0, 5.0)	2.0 (1.0, 3.0)	2.0 (1.0, 3.0)	2.0 (1.0, 4.0)
Household composition (%)						
Couple (with or without children)	70 (22.7)	116 (27.2)	71 (26.6)	56 (25.8)	98 (30.0)	58 (28.9)
Single-parent home	53 (17.2)	85 (20.0)	82 (30.7)	39 (18.0)	68 (20.8)	59 (29.4)
Single (living alone or with others)	185 (60.1)	225 (52.8)	114 (42.7)	122 (56.2)	161 (49.2)	84 (41.8)
Household educational level (%)						
Secondary level or less	241 (78.2)	269 (63.1)	174 (65.2)	161 (74.2)	198 (60.6)	123 (61.2)
Post-secondary studies	67 (21.8)	157 (36.9)	93 (34.8)	56 (25.8)	129 (39.4)	78 (38.8)
Annual household income (%)						
≤CAN$14.999$	208 (67.5)	298 (70.0)	131 (49.1)	121 (57.1)	193 (61.5)	88 (44.9)
≥CAN$15.000$	100 (32.5)	128 (30.0)	136 (50.9)	91 (42.9)	121 (38.5)	108 (55.1)
Length of FB use time before the study, median (IQR)	1.0 (1.0, 3.0)	2.0 (1.0, 3.0)	3 (1.0, 4.0)	1.0 (1.0, 3.0)	2.0 (1.0, 3.0)	3.0 (1.0, 4.0)
Community organization size, median (IQR)	14.0 (9.0, 22.0)	14.0 (8.0, 24.0)	15.0 (8.0, 23.0)	13.0 (9.0, 18.0)	14.0 (8.0, 24.0)	15.0 (8.0, 22.0)
Setting (%)						
Urban	113 (36.7)	326 (76.5)	131 (49.1)	66 (30.4)	246 (75.2)	96 (47.8)
Suburban	143 (46.4)	66 (15.5)	41 (15.4)	116 (53.5)	53 (16.2)	30 (14.9)
Rural	52 (16.9)	34 (8.0)	95 (35.6)	35 (16.1)	28 (8.6)	75 (37.3)
COVID-19 measures (%)						
Non	—	—	—	93 (42.9)	156 (47.7)	121 (60.2)
Yes	—	—	—	124 (57.1)	171 (52.3)	80 (39.8)

### AFSU Profiles and Trends of Food Donation Use

Compared to the overall mean change in food donation use at the end of 12 months of follow-up among FB-exclusive-users, FB+F&V-market-users were more likely to continue using food donations (OR: 1.24; 95% Crl: 0.49, 3.12) and multiple/diverse-AFS-users were less likely to continue using donations (OR: 0.87; 95% Crl: 0.31, 2.42), but the posterior distribution of both estimates included the null value (i.e., one). Although the trends of food donations were influenced by age and setting at baseline, as well as major life events and household income at *t*_1_, only an income ≥ CAN$15.000 at the follow-up showed an indication of decreased likelihood in food donation use.

**FIGURE 3:**
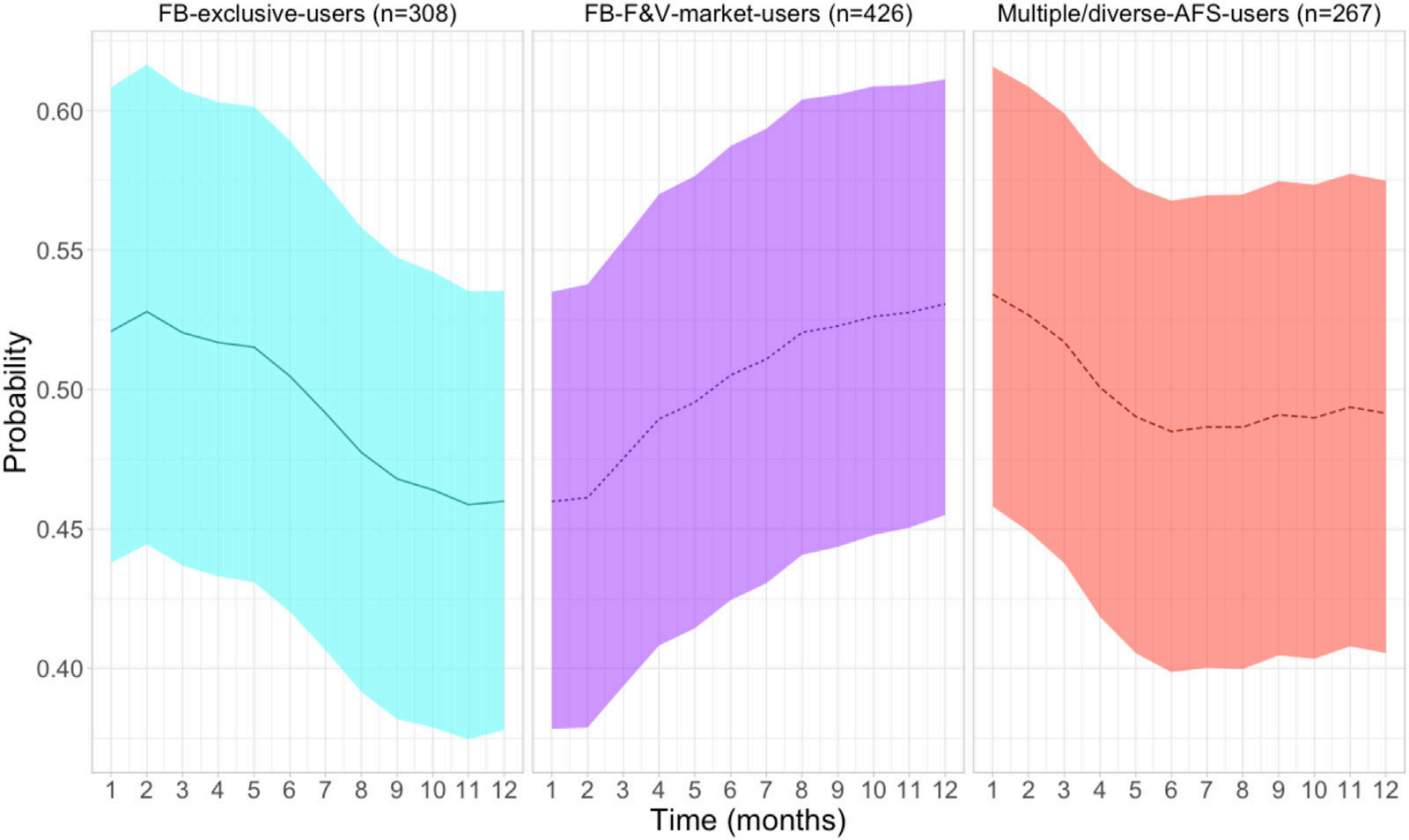
Trends (estimated probabilities and 95% CrIs) of food donation use by alternative food source utilization profiles among newly enrolled food bank users in Quebec, Canada (2018–2021).

The mean random effects coefficients of the interaction between AFSU profile and time on the food donation use by month are available in Supplementary Table S5. The posterior mean distribution of the interaction between AFSU profile and time, which represents the residual trends of food donation use after accounting for other variables, showed an important temporal autocorrelation of food donation use over the time (Rho: 0.76; 95% Crl: 0.76, 0.87). Figure 3 shows that the mean trends of food donation use were different across AFSU profiles. While FB-exclusive-users had a rapid downward mean trend of food donation use after 5 months of the study, multiple/diverse-AFS-users had a flatter trend afterwards. In contrast, FB+FV-market-users presented an upward trend during the study period. However, the posterior mean distribution of the monthly probabilities of food donation use overlapped. The results of the unweighted and ICPW-weighted models of food donation use are presented in Table 2. Adding ICPW to the unweighted model improved the model fit and decreased some credible intervals but did not change the estimates.

**TABLE 2:** Odds ratios for the effect of alternative food source utilization profiles on monthly use of food donations among newly enrolled food bank users in Quebec, Canada (2018–2021).

	Unweighted		IPCW	
	OR	CrI	OR	CrI
Alternative Food Source Utilization Profiles				
FB-exclusive-users	1.00	Reference	1.00	Reference
FB+F&V-market-users	1.26	0.45; 3.52	1.24	0.48; 3.19
Multiple/diverse-AFS-users	0.84	0.27; 2.64	0.87	0.30; 2.49
Age	1.10	1.07; 1.14	1.09	1.06; 1.13
Gender				
Female	1.00	Reference	1.00	Reference
Male	1.31	0.57; 3.01	1.29	0.60; 2.78
Race				
White	1.00	Reference	1.00	Reference
Non-white	0.92	0.25; 3.30	0.95	0.29; 3.10
Country of birth				
Canadian	1.00	Reference	1.00	Reference
Non-Canadian	1.82	0.46; 7.19	1.76	0.50; 6.22
Mental health				
Baseline	1.04	1.00; 1.08	1.04	1.00; 1.07
T1	0.97	0.93; 1.00	0.97	0.94; 1.00
Physical health				
Baseline	1.56	0.45; 5.40	1.49	0.48; 4.67
T1	1.06	0.58; 1.93	1.06	0.61; 1.83
Major life events				
Two or less events	1.00	Reference	1.00	Reference
Three or more events, baseline	0.94	0.40; 2.20	0.94	0.43; 2.04
Three or more events, T1	3.60	1.58; 8.30	3.23	1.51; 6.96
Household composition				
Couple (with or without children)	1.00	Reference	1.00	Reference
Single parent-family	0.50	0.17; 1.48	0.54	0.20; 1.45
Single	0.43	0.16; 1.16	0.47	0.19; 1.17
Household educational level				
Secondary level or less	1.00	Reference	1.00	Reference
Post-secondary studies	1.22	0.52; 2.87	1.20	0.55; 2.64
*Annual household income*				
≤CAN$14.999$	1.00	Reference	1.00	Reference
≥CAN$15.000$, baseline	0.87	0.36; 2.12	0.89	0.39; 2.01
≥CAN$15.000$, T1	0.14	0.06; 0.34	0.17	0.08; 0.37
Length of FB use time before the study				
One or less than 1 month	1.00	Reference	1.00	Reference
Two or more months	1.07	0.37; 3.12	1.06	0.40; 2.84
Size of community organization	0.87	0.52; 1.46	0.88	0.54; 1.42
Setting				
Urban	1.00	Reference	1.00	Reference
Suburban	3.06	1.14; 8.31	2.80	1.13; 7.02
Rural	6.38	2.01; 20.65	5.40	1.86; 15.84
COVID measures				
Non	1.00	Reference	1.00	Reference
Yes	1.34	0.49; 3.73	1.33	0.52; 3.41

### AFSU Profiles and Trends of Other Food-Related CB-SAP Use

Given the small proportion of participants using other food-related CB-SAPs (18% adding up all programs) and the fact that the utilisation of these programs did not change during the study, the models including the interaction between AFSU profiles and time with a first-order autoregressive structure did not result in a good fit. We selected the model without interaction, but still with random effects for user and time with an autoregressive structure because it presented the best fit. The ORs and 95% Cr.Int for the effect of AFSU profiles on other food-related CB-SAPs are shown in Table 3. Compared to FB-exclusive-users, the overall mean of the use of other food-related CB-SAPs was more likely to decrease (OR: 0.77; 95% Crl: 0.24, 2.46) among FB-FV-market- users and more likely to increase among multiple/diverse-AFS-users (OR: 2.39; 95% Crl: 0.67, 8.67). However, the posterior distribution of both estimates includes one indicating the absence of differences in the use of other food-related CB-SAPs across profiles. Age, setting, the length of FB use time before the study, and COVID-19 measures gave an indication of decreased likelihood in the use of other food-related CB-SAPs. The IPCW improved the model fit, improving precision for some credible intervals and estimates.

**TABLE 3:** Odds ratios for the effect of alternative food source utilization profiles on monthly use of other food-related community-based social assistance programs among newly enrolled food bank users in Quebec, Canada (2018–2021).

	Unweighted		IPCW	
	OR	CrI	OR	CrI
Alternative Food Source Utilization Profiles				
FB-exclusive-users	1.00	Reference	1.00	Reference
FB+F&V-market-users	0.76	0.21; 2.70	0.77	0.24; 2.46
Multiple/diverse-AFS-users	2.82	0.71; 11.61	2.39	0.67; 8.67
Age	1.12	1.07; 1.18	1.11	1.06; 1.16
Gender				
Female	1.00	Reference	1.00	Reference
Men	0.46	0.16; 1.29	0.50	0.19; 1.30
Race				
White	1.00	Reference	1.00	Reference
Non-white	0.45	0.09; 2.30	0.51	0.11; 2.24
Country of birth				
Canadian	1.00	Reference	1.00	Reference
Non-Canadian	2.57	0.45; 15.23	2.24	0.45; 11.36
Mental health				
Baseline	1.01	0.96; 1.05	1.01	0.97; 1.05
Time1	0.96	0.91; 1.00	0.96	0.92; 1.00
Physical health				
Baseline	1.42	0.30; 6.84	1.36	0.33; 5.74
Time1	1.96	0.95; 4.57	1.78	0.91; 3.87
Major life events				
Two or less events	1.00	Reference	1.00	Reference
Three or more events, baseline	6.72	2.23; 21.67	5.26	1.92; 15.09
Three or more events, T1	1.46	0.51; 4.19	1.36	0.52; 3.56
Household composition				
Couple (with or without children)	1.00	Reference	1.00	Reference
Single parent-family	0.54	0.13; 2.10	0.61	0.17; 2.14
Single	0.66	0.18; 2.32	0.72	0.23; 2.30
Household educational level				
Secondary level or less	1.00	Reference	1.00	Reference
Post-secondary studies	1.40	0.47; 4.15	1.33	0.49; 3.60
Annual household income				
≤CAN$14.999$	1.00	Reference	1.00	Reference
≥CAN$15.000$ t0	0.67	0.21; 2.04	0.73	0.26; 2.04
≥CAN$15.000$ t1	0.80	0.27; 2.36	0.79	0.29; 2.15
Length of FB use time before the study				
One or less than 1 month	1.00	Reference	1.00	Reference
Two or more months	0.17	0.04; 0.68	0.22	0.06; 0.78
Size of community organization	0.83	0.43; 1.59	0.86	0.47; 1.56
Setting				
Urban	1.00	Reference	1.00	Reference
Suburban	0.04	0.01; 0.15	0.06	0.02; 0.19
Rural	0.11	0.02; 0.47	0.14	0.04; 0.55
COVID measures				
Non	1.000	Reference	1.000	Reference
Yes	0.15	0.04; 0.55	0.19	0.05; 0.63

## Discussion

Our findings indicate that the trends of food donation use during the first year after FB enrollment varies according to AFSU profiles of *de novo* new FBs users, whereas the average trend of other food-related CB-SAPs use did not vary across profiles. These results These results are in line with studies suggesting that FB-users develop diverse strategies, including using different AFSs, to feed themselves and their households [9, 11, 13, 15, 16]. They confirm that new FB-users use food donations in different ways over the time [18–22], which means that this program may have distinct effects on FB-users’ lives. Likewise, consistent with a prior study conducted in Montréal showing that newly registered FB-users in Montréal who used other food-related CB-SAPs were different from those who only used food donations [12], Our findings suggest that people who used these programs before the study baseline, mostly multiple/diverse-AFS-users, were those who continued using them during the 12 months of the study. However, we cannot rule out any association between AFSU profiles and the use of other food-related CB-SAPs.

Our research adds to the body of literature suggesting that some coping strategies may become adapting strategies integrated in the activities of food-insecure households [15, 23, 42] by showing that the use of food donations as a short-term coping strategy may contribute to FB-users’ adaptability in diverse forms according to their needs and capabilities. For instance, the rapid downward mean trend of food donation use among FB-exclusive-users after 5 months of the study may be explained by the fact that some FB-exclusive-users may start using F&V-markets to cope with the limited supply of fruit and vegetables in FBs [2]. Thus, some FB-exclusive-users became FB+F&V-market or multiple/diverse-AFS users during the study, which may also explain the upward trend of FB+F&V-market-users, although we did not formally test this phenomenon. In contrast, the flat mean trend of food donation use observed (in the second half of the follow-up period) among multiple/diverse-AFS-users may indicate that even if they have more opportunities to access food when they start visiting FBs, and their usage of food donations tends to diminish in the first months, some of them continue using this program to meet some of their needs or to save money. This is in line with qualitative studies suggesting that some FB-users use food donations not as a means to address short-term hunger, but as a means of freeing up income for other purposes over time [42, 43]. Consistently, although the likelihood of using food donations seems to decrease with income ≥ CAN$15.000 at t_1_ (post baseline), we did not find great heterogeneity in income distribution, as most participants reported very low incomes in both waves.

Consequently, it seems that the boundaries between using food donations as coping and adapting strategies blur in a global, complex, and dynamic process. In this process FB-users are constantly changing and bargaining in response to several drivers (e.g., food sources availability and sociocultural differences across settings) interacting with their capacities and adaptability through time and space. Thus, it is highly probable that multiple/diverse-AFS-users improve their food security status quickly than exclusively relying on FBs. However, empirical testing is needed.

### Strengths and Limitations

Although our study uses a robust methodology there are some limitations. First, the small sample size can impact the precision of our estimates, especially for rural settings. Likewise, the assessment of AFSU and time interaction could be underpowered. The assessment of statistical interactions requires larger sample sizes and therefore, the lack of statistical significance in this context should not be misunderstood as a lack of effect. Second, given that the *Pathway Study* includes only *de novo* FB-users in four regions of Quebec, interpretation is intended for this province. Further research is needed on this topic to explore differences in trends of food donation and other food-related CB-SAP use among FB-users in other regions of the country. Third, we did not consider the usage frequency, or the quality and size of FB parcels when modeling the trends, although these may influence the use of CB-SAPs. However, the frequency of food donations use is one of the key variables used to identify AFSU profiles and therefore, partially accounted for. Fourth, our results might be affected by recall bias because information related to programs use and time-variant covariates in the previous 12 months was assessed using a self-reported questionnaire at *t*1. Fifth, the COVID-19 pandemic could have affected our study in various ways. For instead, living during the pandemic could have affected the validity of the LTE-Q, potentially considering it as a main stressor, an overall modifier, or an indicator of stressors, thus participants’ perception of stress may have changed during this period. It could also have exacerbated the reliance on food donations during this period [44–46]. Even though we included a variable to account for these consequences, we were unable to control for all of them.

Despite these limitations, to the best of our knowledge, this study is the first to assess the influence of AFSU on the use of food donations and other food-related CB-SAPs over time among *de novo* FB users. An important strength of this study relates to use of a well-characterized cohort of new FB-users in urban, semiurban, and rural areas with detailed follow-up information. The advantage of using repeated measures since the beginning of FB enrollment in diverse community organizations located in different settings, allowed the Bayesian modelling of data for within-individual variations in the use of food donations and other food-related CB-SAPs. This permitted considering setting and community organization differences, and potential clustering, while estimating trends of use addressing temporal autocorrelation and accounting for censoring via IPCW ensuring robustness of our estimates.

### Conclusion

This study highlights the diverse trends of food donation use among new FB-users, indicating that this program has different meaning for FB-users depending on their background and needs. Hence, FB-users’ responses to food shortages should be understood as dynamic processes. For some, food donation use serves as a short-term strategy to cope with food insecurity, while for others, the use of this program evolves into a long-term strategy. This transformation is contingent upon their AFS-utilization profile and their adaptability to evolving socioeconomic conditions. Recognizing these processes and documenting changes over time can help to predict FB-users’ pathways and indicate appropriate ways to improve access to food among this population.
