# Supplementary material for: Characterizing Trends in the Use of Food Donations and Other Food-Related Community-Based Social Assistance Programs in a Cohort of New Food Bank Users in Quebec, Canada
Source: Int J Public Health. 2024 Feb 9;69:1605833. doi: 10.3389/ijph.2024.1605833 (PMC10884234; doi:10.3389/ijph.2024.1605833)
Supplement: Supplementary file 1 [file DataSheet1.docx]

| **Table S1. Model fit of food donation use** | | | | | | |
| --- | --- | --- | --- | --- | --- | --- |
| **Model** | **DIC** | | **WAIC** | | **logLik** | |
| RI (iid) setting, interaction (time-AFSU), and ID | 5556.79 | | 5320.14 | | -3088.19 | |
| RI (iid) ID + RI (ar1) interaction (time-AFSU) | 5544.58 | | 5313.14 | | -3077.54 | |
| RI (iid) setting and ID + RI (ar1) interaction (time-AFSU) | 5541.23 | 5307.91 | | -3078.1 | |  |
| RI (iid) setting, time, and ID + RI (ar1) interaction (time-AFSU) | 5553.11 | | 5319.2 | | -3090.79 | |
| RI (iid) time and ID + RI (ar1) interaction (time-AFSU) | 5554.32 | | 5323.74 | | -3090.53 | |
| RI (iid) time and ID + RI (ar1) interaction (time-AFSU) +IPCW | 4488.23 | | 4031.03 | | -2552.77 | |

*RI=Random intercept

| **Table S2. Model fit of the use of other food-related community-based assistance programs** | | | |
| --- | --- | --- | --- |
| **Model** | **DIC** | **WAIC** | **logLik** |
| RI (iid) ID + RI (ar1) interaction (time-AFSU) | 5764.15 | 6471.43 | -1601.45 |
| RI (iid) setting and ID + RI (ar1) time | 5708.16 | 6319.35 | -1610.71 |
| RI (iid) ID and RI (ar1) time | 5702.04 | 6351.12 | -1604.31 |
| RI (iid) ID + RI (ar1) time and setting | 5668.46 | 6228.13 | -1609.89 |
| RI (iid) setting and ID + RI (ar1) time +IPCW | 4373.37 | 4010.09 | -1355.76 |
| RI (iid) ID and RI (ar1) time + IPCW | 4391.51 | 4049.31 | -1359.03 |

*RI=Random intercept

| **Table S3. Distribution of missing data** | | |
| --- | --- | --- |
| **Variable** | **Baseline (2018-2020)** | **Wave 1 (2019-2021)** |
|  | **n= 1001 (%)** | **n=745 (%)** |
| **Age,** y, mean (SD) | 41.03 (12.05) |  |
| ***Gender**** |  |  |
| Male | 389 (38.9) |  |
| Female | 610 (60.9) |  |
| Missing | 2 (0.2) |  |
| ***Household educational level****** |  |  |
| Secondary level or less | 684 (68.3) |  |
| Post-secondary studies | 317 (31.7) |  |
| ***Annual household income t0**** |  |  |
| ≤ 14.999$ | 605 (60.5) | 402 (40.2) |
| ≥ 15.000$ | 364 (36.4) | 320 (32.0) |
| Missing | 32 (3.2) | 23 (3.10) |
| ***Length of FB use time before the study*** |  |  |
| Between 3 and 6 months | 374 (37.4) |  |
| ≤ 2 months | 627 (62.6) |  |
| ***Setting*** |  |  |
| Urban | 570 (56.9) |  |
| Suburban | 250 (25.0) |  |
| Rural | 181 (18.1) |  |
| ***Alternative food source utilization profile*** |  |  |
| FB-exclusive-users | 308 (30.8) |  |
| FB+F&V-market-users | 415 (41.4) |  |
| Multiple/diverse-AFS-users | 267 (26.7) |  |
| Missing | 11 (1.1) |  |
| ***Major life events,*** *y, mean (SD)* | 3.3 (2.03) | 2.43 (1.82) |
| Missing |  | 1 |
| ***Collective kitchen*** |  |  |
| Yes | 107 (10.7) | 72 (9.7) |
| No | 865 (86.4) | 671 (90.1) |
| Missing | 29 (2.9) | 1 (0.1) |
| ***Community garden*** |  |  |
| Yes | 29 (2.9) | 25 (3.4) |
| No | 972 (92.1) | 716 (96.1) |
| Missing |  | 4 (0.5) |
| ***Food-purchasing group*** |  |  |
| Yes | 55 (5.5) | 31 (4.2) |
| No | 946 (94.5) | 713 (95.7) |
| Missing |  | 1 (0.1) |
| ***Food sales service*** |  |  |
| Yes | 73 (7.3) | 28 (3.8) |
| No | 928 (92.7) | 716 (96.1) |
| Missing |  | 1 (0.1) |
| ***Community meals*** |  |  |
| Yes | 163 (16.3) | 75 (10.1) |
| No | 838 (83.7) | 669 (89.8) |
| Missing |  | 1 (0.1) |
| ***Class or workshops on cooking or food related topics*** |  |  |
| Yes | 40 (4.0) | 25 (3.4) |
| No | 961 (96.0) | 719 (96.5) |
| Missing |  | 1 (0.1) |

| **Table S4. Descriptive statistics for retained and not retained for analysis, Pathways Study, Quebec, Canada, 2018-2022** | | |
| --- | --- | --- |
| Characteristics* | Censored  (n=256)*** | Retained (n=745)** |
| **Age,** *y, median (IQR)* | 39.0 (30.0, 50.0) | 41.0 (32.0, 52.0) |
| ***Gender,*** % (95% CI) |  |  |
| Male | 50.4 (44.1; 56.7) | 35 (31.6; 38.5) |
| Female | 49.6 (43.3; 55.9) | 65 (61.5; 68.4) |
| ***Country of birth****, % (95% CI)* |  |  |
| Other | 15.6 (11.4; 20.7) | 25 (21.9; 28.2) |
| Canada | 84.4 (79.3; 88.6) | 75 (71.8; 78.1) |
| ***Race,*** *% (95% CI)* |  |  |
| Other | 19.9 (15.2; 25.3) | 26.8 (23.6; 30.1) |
| White | 80.1 (74.7; 84.8) | 73.2 (69.9; 76.4) |
| ***Mental health****, mean (SD)* | 40.7 (29.8, 49.3) | 41.0 (32.0, 49.9) |
| ***Physical health,*** *median (IQR)* | 49.1 (36.0, 56.70 | 48.5 (35.5, 56.1) |
| ***Major life events ,*** *median (IQR)* | 3.0 (2.0,5.0) | 3.0 (2.0, 5.0) |
| ***Household composition****, % (95% CI)* |  |  |
| Couple (with or without children) | 17.6 (13.1; 22.8) | 28.5 (25.2; 3.18) |
| Single-parent home | 21.1 (16.3; 26.6) | 22.3 (19.3; 2.54) |
| Single (living alone or with others) | 61.3 (55.1; 67.3) | 49.3 (45.6; 52.9) |
| ***Household educational level***, % (95% CI) |  |  |
| Secondary level or less | 78.9 (73.4; 83.7) | 64.7 (61.1; 68.1) |
| Post-secondary studies | 21.1 (16.3; 26.6) | 35.3 (31.9; 38.9) |
| ***Annual household income***, % (95% CI) |  |  |
| ≤ 14.999$ | 68.8 (62.7; 74.4) | 61.9 (58.3; 65.4) |
| ≥ 15.000$ | 31.3 (25.6; 37.3) | 38.1 (34.6; 41.7) |
| ***Length of FB use time before the study,*** *median (IQR)* | 2.0 (1.0, 3.0) | 2.0 (1.0, 3.0) |
| ***Size of the community organization****, median (IQR)* | 17.0 (12.0, 28.0) | 14.0 (8.0,22.0) |
| ***Setting***, % (95% CI) |  |  |
| Rural | 16.8 (12.4; 22) | 18.5 (15.8; 21.5) |
| Suburban | 19.9 (15.2; 25.3) | 26.7 (23.6; 30) |
| Urban | 63.3 (57.1; 69.2) | 54.8 (51.1; 58.4) |
| ***Alternative food sources utilization profile*** |  |  |
| FB-exclusive users | 35.5 (2.97; 26.6) | 29.1 (25.9; 32.5) |
| FB and FV-market users | 38.7 (32.7; 44.9) | 43.9 (40.3; 47.5) |
| Multi-AFS users | 25.8 (20.5; 31.6) | 27 (23.8; 30.3) |
| * Data were drawn from the baseline (collected in 2018-2020); ** Includes participants with complete data in the two waves; *** Includes participants with data in baseline only | | |

**Table S5. Mean random effects coefficients and Crl of the interaction between alternative food source utilization profile and time on the food donation use.**

|  | FB-exclusive (n=308) | | FB-FV market (n=426) | | Multiple\diverse-AFS (n=267) | |
| --- | --- | --- | --- | --- | --- | --- |
| Month | Mean | Crl (95%) | Mean | Crl (95%) | Mean | Crl (95%) |
| 1 | 0.08 | -0.25; 0.44 | -0.16 | -0.50; 0.14 | 0.14 | -0.17; 0.47 |
| 2 | 0.11 | -0.22; 0.48 | -0.16 | -0.49; 0.15 | 0.11 | -0.20; 0.44 |
| 3 | 0.08 | -0.25; 0.44 | -0.10 | -0.43; 0.22 | 0.07 | -0.25; 0.40 |
| 4 | 0.07 | -0.27; 0.42 | -0.04 | -0.37; 0.28 | 0.00 | -0.33; 0.33 |
| 5 | 0.06 | -0.28; 0.41 | -0.02 | -0.35; 0.31 | -0.04 | -0.38; 0.29 |
| 6 | 0.02 | -0.32; 0.36 | 0.02 | -0.30; 0.35 | -0.06 | -0.41; 0.27 |
| 7 | -0.03 | -0.38; 0.30 | 0.04 | -0.28; 0.38 | -0.05 | -0.40; 0.27 |
| 8 | -0.09 | -0.44; 0.23 | 0.08 | -0.24; 0.42 | -0.05 | -0.41; 0.28 |
| 9 | -0.13 | -0.48; 0.19 | 0.09 | -0.23; 0.43 | -0.04 | -0.39; 0.30 |
| 10 | -0.14 | -0.49; 0.17 | 0.10 | -0.23; 0.44 | -0.04 | -0.39; 0.30 |
| 11 | -0.17 | -0.57; 0.14 | 0.11 | -0.20; 0.44 | -0.03 | -0.37; 0.30 |
| 12 | -0.16 | - 0.50; 0.14 | 0.12 | -0.18; 0.45 | -0.03 | -0.38; 0.30 |
